# Treatment failure in a dog with acute *Babesia canis* infection using lower-range therapeutic doses of imidocarb dipropionate

**DOI:** 10.1007/s00436-026-08664-8

**Published:** 2026-03-17

**Authors:** Ingo Schäfer, Vanessa Baitis, Christina Strube, Kriti Jindal, Elisabeth Müller, Torsten J. Naucke, Andreas Moritz

**Affiliations:** 1 Laboklin GmbH and Co. KG, ²Kleintierpraxis Weissbach GbR, Bad Kissingen, Steina, Germany; 2 Institute for Parasitology, Centre for Infection Medicine, University of Veterinary Medicine Hannover, Hanover, Germany; 3 Small animal clinic – Internal Medicine, Department of Veterinary Medicine, Justus-Liebig-University Giessen, Giessen, Germany; 4 Centre for Infection Medicine, University of Veterinary Medicine Hannover, Hanover, Germany

**Keywords:** Vector-borne infection, Babesiosis, Imidocarb dipropionate, Therapy

## Abstract

Canine babesiosis caused by *Babesia canis* is considered an emerging disease in Central and Eastern European countries, including Germany. Imidocarb dipropionate is recommended for treatment of acute *B. canis* infections. A nine-year-old, male intact Golden Retriever living in Saxony, Germany, was presented in November 2025 due to lethargy, inappetence, vomiting, fever (39.6 °C), and darkened urine to a small animal veterinary practice. Ectoparasite prophylaxis was discontinued three months before the onset of clinical signs. An acute *B. canis* infection was diagnosed based on microscopic detection of large merozoites in peripheral blood smears, followed by *Babesia* spp. PCR and sequencing. Hematological examination revealed marked thrombocytopenia, mild non-regenerative anemia, lymphopenia, and eosinopenia. The dog remained PCR positive after two imidocarb dipropionate injections (2.0 mg/kg bodyweight and 5.0 mg/kg bodyweight s.c.), and hematological abnormalities were still present. Prednisolone (2.0 mg/kg bodyweight s.c., continued orally) was applied, after which merozoites were still detected in peripheral blood smear analysis, so prednisolone was discontinued. After two further imidocarb dipropionate injections (9.4 mg/kg bodyweight s.c. each), the dog presented as clinically healthy, and a subsequent PCR testing was negative. From this report, different measures for dogs suffering from an acute* B. canis* infections can be derived: Dogs should be treated with a high dosage of imidocarb dipropionate of 6.6 mg/kg bodyweight as approved by the FDA, and the treatment success should be monitored by PCR 14 days post treatment. If treatment fails after two administrations at either dose of imidocarb dipropionate, i.e. if PCR remains positive, it is recommended to verify a *B. canis* infection by molecular species differentiation, e.g. via sequencing. Furthermore, dogs with treatment failure should be screened for coinfections and underlying comorbidities, which may complicate the course of an acute *B. canis* infection. Due to the authors’ personal experience, application of corticosteroids, even in non-immunosuppressive dosages, should be avoided, especially if blood transfusions are not necessary and/or marked hemolytic anemia is absent. Furthermore, this report highlights the importance of efficient year-round tick control, even in winter months.

## Introduction

*Babesia canis* is a haemoprotozoal pathogen transmitted by *Dermacentor reticulatus* ticks. First autochthonous *B. canis* infections in dogs without travel history were reported in Germany in the 1990s (Zahler et al. 2000, Glaser and Gothe 1998). Meanwhile, such infections are observed in a considerable number of German dogs (Weingart et al. 2023, Schäfer et al. 2023, Eisenecker et al. 2025, Hohnhorst et al. 2025), mainly linked to the expansion of the vector *D. reticulatus* due to climate change, changes in land use, and increased import and travel of pets especially from eastern and southeastern European countries (Diakou 2024, Mierzejewska et al. 2017, Gray et al. 2009). As *D. reticulatus* ticks are meanwhile distributed in each German federal state and are active throughout the whole year, including the winter months, infections with *B. canis* must be considered in dogs with corresponding clinicopathological abnormalities as a potential differential diagnosis year-round, especially in dogs with thrombocytopenia (Weingart et al. 2023, Schäfer et al. 2023, Hohnhorst et al. 2025, Drehmann et al. 2020, Springer et al. 2022, Probst et al. 2023, Probst et al. 2023). Especially the German federal states of Berlin/Brandenburg, Saxony, Saxony-Anhalt, and Saarland as well as the Ruhr area and the Rhine-Main area were recently classified as high-risk areas for *B. canis* infections in dogs (Weingart et al. 2023, Schäfer et al. 2023, Eisenecker et al. 2025, Seibert et al. 2022).

Acute *B. canis* infections may vary from subclinical infections to severe and life-threatening disease (Eisenecker et al. 2025, Hohnhorst et al. 2025). Dogs with acute *B. canis* infections in Germany are most often presented with unspecific clinical signs as lethargy, fever, and inappetence (Weingart et al. 2023, Eisenecker et al. 2025, Seibert et al. 2022), whereas pigmenturia is recently reported in only about one quarter of dogs with acute infections (Eisenecker et al. 2025). Most often marked thrombocytopenia is the most common hematological abnormality, followed by most often mild anemia, and leukopenia (Weingart et al. 2023, Eisenecker et al. 2025, Seibert et al. 2022). Acute *B. canis* infections are accompanied by an acute-phase reaction with elevated C-reactive protein (CRP) and decreased albumin (Hohnhorst et al. 2025). Life-threatening complications can include systemic inflammatory response syndrome (SIRS), disseminated intravascular coagulopathy (DIC), acute renal failure, multi-organ dysfunction syndrome (MODS), acute respiratory distress syndrome with pulmonary edema, and cerebral babesiosis (Dubova et al. 2023, Matijatko et al. 2010, Adaszek et al. 2012, Zygner et al. 2021).

Diagnosis of acute *B. canis* infections in dogs is primarily based on positive polymerase chain reaction (PCR) results from venous and/or capillary blood, which shows high sensitivity and specificity. Microscopic detection of merozoites in erythrocytes is less sensitive and specific, but, especially with smears from capillary blood, useful as a rapid and cost-effective in-house methodology (Solano-Gallego et al. 2016, Böhm et al. 2006, Schetters 2019). However, negative results should be confirmed by PCR, and positive results allow differentiation of the various *Babesia* spp. by sequencing. Positive *Babesia* spp. antibody serology demonstrates past pathogen contact but does not prove an acute *B. canis* infection (Solano-Gallego et al. 2016, Schaarschmidt et al. 2006). The higher the *Babesia* spp. antibody levels at the time of infections, the less severe are the clinicopathological abnormalities and the lower the infection intensity, indicating protective antibodies (Hohnhorst et al. 2025).

Two subcutaneous or intramuscular injections of 6.6 mg/kg bodyweight (BW) imidocarb dipropionate 14 days apart are approved by the Food and Drug Administration (FDA) in the USA under NADA 141 − 071 (FDA). In Europe, imidocarb dipropionate is labelled with a therapeutic dosage of 0.25 ml/10 kg BW (i.e., 3.0 mg/kg) and a prophylactic dosage of 0.5 ml/10 kg BW (i.e., 6.0 mg/kg BW). The administration of glucocorticoids and/or doxycycline in combination with imidocarb dipropionate with the aim to reduce the acute-phase response and immunopathological reactions is controversially discussed (Nevidnyk-Pravda and Ushakova 2025, Vercammen et al. 1996). If the disease is diagnosed early, prognosis is generally good. Dog mortality rates due to acute *B. canis* infections vary from 1.4% in southwestern up to 20% in northeastern and central Europe, with 7–13% in Germany, especially in dogs without any stays abroad (Weingart et al. 2023, Eisenecker et al. 2025, Seibert et al. 2022).

## Materials and methods

Real-time PCR testing for *Babesia* spp. on EDTA blood followed by species differentiation via sequencing was performed at IDEXX Laboratories (Kornwestheim, Germany) on EDTA blood and at the Laboklin laboratory (Bad Kissingen, Germany) retrospectively on already purified DNA by a piroplasm PCR (forward primer: 5′-AAT ACC CAA TCC TGA CAC AGG G-3′; reverse primer: 5′-TTA AAT ACG AAT GCC CCC AAC-3′, based on Olmeda et al. (Olmeda et al. 1997)) followed by Sanger sequencing). Additionally, real-time PCR testing for *Anaplasma* spp., *Ehrlichia* spp, and *Hepatozoon* spp. was conducted at IDEXX laboratories on EDTA blood (Kornwestheim, Germany). A complete blood count on EDTA blood (IDEXX ProCyte DX Hematology Analyzer), and a biochemistry profile on serum (IDEXX Catalyst One Chemistry Analyzer) were performed in-house in the small animal veterinary practice on days 0, 9, 10, 21, 26, and 38.

On day 54, a complete blood count on EDTA blood (Sysmex XN-V analyzer, Sysmex Deutschland GmbH, Norderstedt, Germany), a biochemistry profile on serum (Cobas 8000, Roche Deutschland Holding GmbH, Mannheim, Germany), CRP on serum (Gentian Canine CRP Immunoassay, run on Cobas 8000, Roche Deutschland Holding GmbH, Mannheim, Germany), *Babesia* spp. Immunoglobulin G (IgG)-antibody detection on serum (Babesia ELISA Dog, Afosa, Blankenfelde-Mahlow, Germany; > 19 TE positive), and a piroplasm PCR on EDTA blood as described above was performed at the Laboklin laboratory (Bad Kissingen, Germany).

Quantification of the *B. canis* parasitemia was performed retrospectively in the Laboklin laboratory using a droplet digital PCR (ddPCR, Bio-Rad) targeting the *Bc28.1* gene specific for *B. canis* (forward primer: 5′-GCT ACG TCC GTT GAA GCC-3′ (10 µM), reverse primer: 5′-TCA GCG GAA TAA CGT TCA GC-3′ (10 µM), probe: 5′-FAM-AGC CAG TCG ATC TGC TCC TTT AAG CT-BHQ-3′ (2 µM), based on Kivrane et al. (Kivrane et al. 2021)).

### Case report

A nine-year-old, male intact Golden Retriever living in Saxony, Germany, was presented in November 4th, 2025 due to lethargy, inappetence, vomiting, fever (rectal temperature 39.6 °C), and darkened urine in the first small animal veterinary practice. The clinical signs were present for two days. An acute *Babesia* infection, presumably with *B. canis*, was diagnosed due to microscopic detection of large merozoites in the peripheral blood smear. Immediately after diagnosis, 2.0 mg/kg BW imidocarb dipropionate as well as metamizole (unknown dosage) were administered subcutaneously on November 4th, 2025. Additionally, meloxicam (0.2 mg/kg BW) was given to the owner for oral administration at home (Table 1).

**Table 1:** Clinical signs and course of treatment in a nine-year-old, male intact Golden Retriever with an acute *Babesia canis* infection from first presentation (D0) to day 54 (D54)

Day	Clinical signs	RT (in °C)	Treatment	*Babesia canis*
Day − 1	Lethargy and inappetence for 2 days	39.6	Carbesia^®^ (imidocarb dipropionate) 2.0 mg/kg s.c. Metacam^®^ (meloxicam) 0.2 mg/kg per os Novaminsulfon (metamizole) s.c.	Merozoites in peripheral blood smear
Day 0 (first presentation)	Lethargy, hemoglobinuria	39.1	Infusion Sterofundin ISO B. Braun Vet Care 7 ml/kg/h for 9 h i.v.	PCR positive
Day 9	Lethargy, inappetence, hemoglobinuria	39.0	Carbesia^®^ (Imidocarb dipropionate) 5.0 mg/kg s.c. Metapyrin^®^ (metamizole) 31 mg/kg i.m. Infusion Sterofundin ISO B. Braun Vet Care 10 ml/kg for 3 h i.v.	PCR positive Merozoites in peripheral blood smear
Day 10	Lethargy, vomitus	40.1	Infusion Sterofundin ISO B. Braun Vet Care 10 ml/kg for 3 h i.v. Prevomax^®^ (maropitant) 1 mg/kg s.c. Metapyrin^®^ (metamizole) 45 mg/kg i.m. Prednisolone 2 mg/kg s.c., continued p.o. and gradually reduced over 4 days, afterwards discontinued	-
Day 21	Lethargy, no hemoglobinuria	39.4	Imidocarb dipropionate 9.4 mg/kg s.c. Metapyrin^®^ (metamizole) 40 mg/kg s.c.	Merozoites in peripheral blood smear
Day 26	Tachypnoea	-	No medication	-
Day 38	Unremarkable	-	Imidocarb dipropionate 9.4 mg/kg s.c. Doxytab^®^ (doxycycline) 10 mg/kg SID p.o.	PCR positive
Day 54	Unremarkable	38.2	Doxytab^®^ (doxycycline) stopped, no further medication needed	PCR negative No merozoites in capillary blood smear analysis

*i.m.* intramuscular injection, *i.v.* intravenously, *p.o.* per os, RT rectal temperature *s.c.* subcutaneous injection

Day 0 is defined as the time of first presentation to another small animal veterinary practice for a second opinion on November 5th, 2025. During anamnesis, the owner reported that the dog had accompanied him on vacation to Poland five years ago and to the Czech Republic six months ago, in May 2025. Two weeks ahead of the onset of clinical signs, the dog travelled to Bad Salzungen, federal state of Thuringia, Germany, for 12 days (October 6th to 18th, 2025), where there had been increasing press reports of autochthonous cases of canine babesiosis. Ectoparasite prophylaxis was applied from June to September 2025 (Imidacloprid and Flumethrin, Seresto^®^ Halsband für Hunde, Elanco Animal Health GmbH, Monheim, Germany), but discontinued two months before the onset of clinical signs, and tick attachment was not recognized by the owners in the last weeks. The general examination revealed pale mucous membrane and a rectal temperature of 39.1 °C. A marked thrombocytopenia, mild non-regenerative anemia, lymphopenia, eosinopenia, and a mild elevation of urea was observed in hematological and biochemical analyses (Table 2). PCR from EDTA blood and subsequent sequencing revealed a *B. canis* infection, and the level of parasitemia accounted to 1,937,000 parasites/ml blood. PCR testings for other tick-borne pathogens were negative. The dog received an intravenous infusion with Sterofundin ISO B. Braun Vet Care (7 ml/kg BW/h) for nine hours. In the next eight days, the dog was unremarkable for the owners, and the color of the urine normalized.

**Table 2:** Complete blood count, biochemical parameters, level of *Babesia canis* parasitemia, *Babesia* spp. antibodies, and microscopic detection of merozoites in a nine-year-old, male intact Golden Retriever with an acute *Babesia canis* infection

Parameter	Reference interval days 0-38^1,2^	Day 0^1,2^	Day 9^1,2^	Day 10^1,2^	Day 21^1,2^	Day 26^1,2^	Day 38^1,2^	Reference interval day 54^3,4^	Day 54^3,4^
Complete blood count									
RBC (x 10^12^/l)	5.65–8.87	**4.4**	**5.36**	**4.13**	**4.47**	**5.11**	**4.52**	5.5–8.5	5.68
HGB (g/dl)	13.1–20.5	**11.1**	**13.0**	**10.2**	**10.6**	**12.4**	**11.0**	15.0–19.0	**14.4**
HCT(l/l)	0.37–0.62	**0.28**	**0.35**	**0.27**	**0.30**	**0.36**	**0.31**	0.44–0.52	0.45
Ret (x 10^9^ /l)	10.0-110.0	11.0	85.2	59.5	**9.4**	**139.5**	11.8	< 110.0	17.6
WBC (x 10^12^/l)	5.1–16.8	5.2	8.9	7.8	9.4	11.2	5.3	6.0–12.0	6.3
Seg (x 10^9^/l)	3.0-11.6	3.6	8.5	6.9	8.4	7.7	2.6	3.0–9.0	3.6
Lymphs (x 10^9^/l)	1.1–5.1	**0.5**	**0.3**	**0.3**	**0.6**	2.3	1.93	1.0-3.6	2.0
Eos (x 10^9^/l)	0.06–1.23	**0.0**	**0.0**	**0.0**	0.1	0.1	0.1	0.04–0.6	0.3
Monos (x 10^9^/l)	0.2–1.1	1.1	**0.1**	0.5	0.3	1.0	0.7	0.04–0.5	0.4
THR (x 10^9^/l)	148–484	**12**	**44**	**8**	**23**	**93**	**146**	150–500	246
**Biochemistry**									
Total protein (g/dl)	5.2–8.2	6.4	6.7	-	6.6	-	-	5.4–7.5	5.9
Albumin (g/dl)	2.3-4.0	2.5	2.9	-	2.8	-	-	2.5–4.4	3.5
Globulins (g/dl)	2.5–4.5	3.9	3.8	-	3.8	-	-	< 4.5	2.4
Urea	7–27 mg/dl	**35**	18	-	16	-	-	3.3–8.3 mmol/l	6.9
Creatinine	0.5–1.8 mg/dl	1.2	0.6	-	0.7	-	-	< 125.0 mmol/l	60.0
Phosphorus	2.5–6.8 mg/dl	4.1	3.7	-	4.3	-	-	0.7–1.6 mmol/l	1.3
Calcium	7.9–12.0 mg/dl	7.9	8.8	-	8.9	-	-	2.3-3.0 mmol/l	2.5
Potassium (mmol/l)	-	-	-	-	-	-	-	3.5–5.1	4.7
Sodium (mmol/l)	-	-	-	-	-	-	-	140–155	147
Magnesium (mmol/l)	-	-	-	-	-	-	-	0.6–1.3	0.8
Bilirubin	< 0.9 mg/dl	0.7	0.7	-	0.3	-	-	< 3.4 µmol/l	0.7
ALT (U/l)	10–125	79	113	-	47	-	-	< 88	43
AP (U/l)	23–212	148	127	-	147	-	-	< 147	80
AST (U/l)	-	-	-	-	-	-	-	< 51	25
CK (U/l)	-	-	-	-	-	-	-	< 200	84
GLDH (U/l)	-	-	-	-	-	-	-	< 8.0	5.1
G-GT (U/l)	-	-	-	-	-	-	-	< 10.0	3.9
Glucose	70–143 mg/dl	81	116	-	95	-	-	3.05–6.1 mmol/l	5.4
Cholesterol	110–320 mg/dl	258	295	-	254	-	-	3.1–10.1 mmol/l	8.5
Iron (µmol/l)	-	-	-	-	-	-	-	15–45	25
Lipase (U/l)	200-1,800	216	269	-	205	-	-	-	-
DGGR-Lipase (U/l)	-	-	-	-	-	-	-	< 120.0	36.2
CRP (mg/l)	-	-	-	-	-	-	-	< 15.0	4.3
**Parasitology**									
*Babesia canis* parasitemia^5^	0 parasites/ml EDTA blood	1,937,000	5,200	-	-	-	129,000	-	0
Merozoites	-	Yes	Yes	-	Yes	-	-	-	No
*Babesia* spp. antibody ELISA^6^	-	-	-	-	-	-	-	< 19 TE	65.4

^1^Hematology: IDEXX ProCyte DX Hematology Analyzer; ^2^Biochemistry: IDEXX Catalyst One Chemistry Analyzer; ^3^Hematology Laboklin GmbH & Co. KG: Sysmex XN-V analyzer, Sysmex Deutschland GmbH, Norderstedt, Germany; ^4^Biochemistry Laboklin GmbH & Co. KG: Cobas 8000, Roche Deutschland Holding GmbH, Mannheim, Germany; 5droplet digital PCR (Bio-Rad) targeting the *Bc28.1* gene; ^6^Babesia ELISA Dog, Afosa, Blankenfelde-Mahlow, Germany

*ALT* alanine transaminase, *AP* alkaline phosphatase, *AST* aspartate aminotransferase *CK* creatine kinase, *CRP* C-reactive protein, *DGGR-lipase*: 1,2-o-dilauryl-rac-glycero-3-glutaric acid-(6’-methylresorufin) ester lipase, *EDTA* ethylenediaminetetraacetic acid, *ELISA* enzyme-linked immunosorbent assay, *Eos* eosinophilic granulocytes, *G-GT* gamma-glutamyl transferase, *GLDH* glutamate dehydrogenase, *HCT* hematocrit, *HGB* hemoglobin, *Lymphs* lymphocytes, *MCH* mean corpuscular hemoglobin, *MCHC* mean corpuscular hemoglobin concentration, *MCV* mean corpuscular volume, *Monos* monocytes, *RBCs* red blood cells, *Seg* segmented neutrophilic granulocytes, *Ret* reticulocytes, *TE* technical units, *THR* platelets, *WBCs* white blood cells

bolded values = parameters outside the reference intervals

**Fig. 1:**
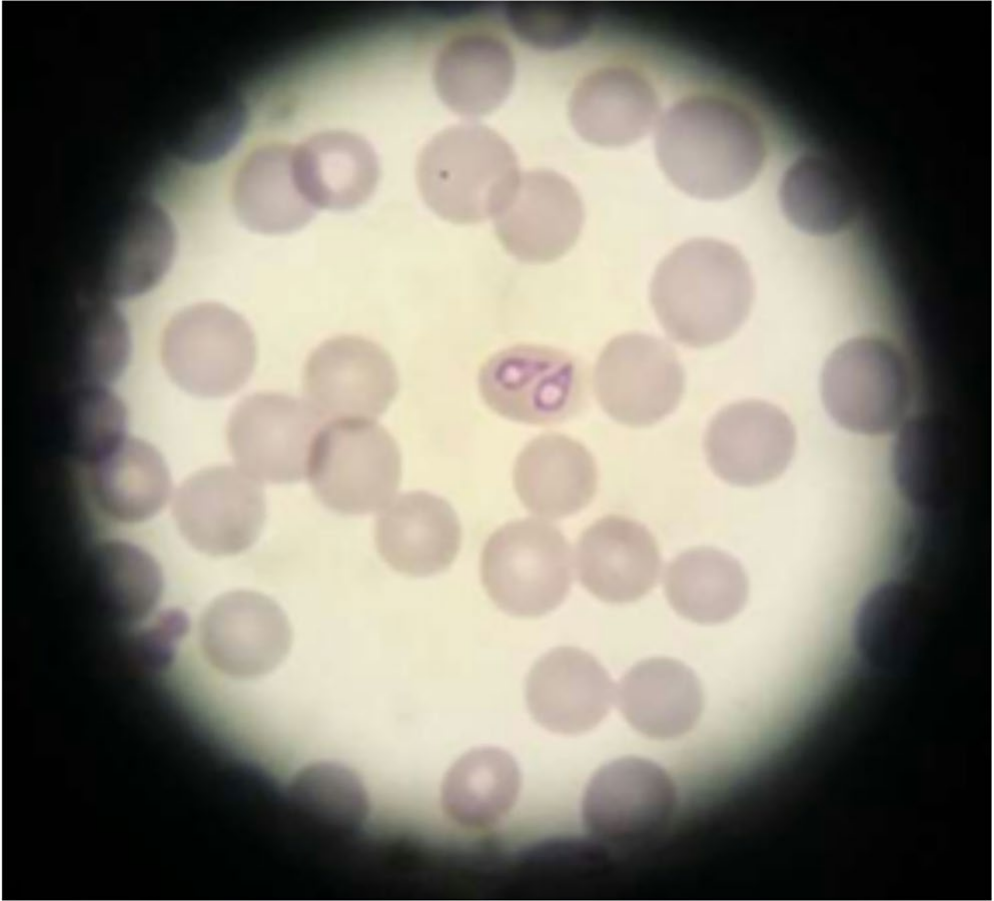
Presence of large *Babesia* merozoites in the peripheral blood of a nine-year-old, male intact Golden Retriever with acute *Babesia canis* infection on day 9 after the first presentation

On day 9, the dog was presented to the small animal veterinary practice again due to recurrence of inappetence and lethargy. In general examination the dog was lethargic with a rectal temperature of 39.0 °C. Additionally, hemoglobinuria was diagnosed. Intraerythrocytic large *Babesia* merozoites were again identified in peripheral blood smear analysis (Fig. 1), and PCR on EDTA blood with subsequent sequencing revealed a level of parasitemia of 5,200 *B. canis* parasites/ml blood. The thrombocytopenia was still marked and the anemia mild, and lymphopenia and eosinopenia were still recognized (Table 2). The biochemical parameters were unremarkable (Table 2). The dog received a second subcutaneous injection of imidocarb dipropionate, this time at a dose of 5.0 mg/kg BW. Furthermore, the dog was given an intramuscular injection of metamizole (31 mg/kg), and intravenous infusion with Sterofundin ISO B. Braun Vet Care (4 ml/kg BW/h for three hours).

The dog was presented again the next day (day 10) with vomitus and fever (40.1 °C). Both thrombocytopenia and anemia worsened (Table 2). Maropitant (1 mg/kg BW s.c.), prednisolone (2 mg/kg BW s.c.), and another intravenous infusion with Sterofundin ISO B. Braun Vet Care (4 ml/kg BW/h for three hours) was given (Table 1). Prednisolone (2.0 mg/kg BW) was additionally applied orally and gradually reduced over four days, and then discontinued.

The dog was recovering and in good condition until day 21, when it was presented once again with lethargy. The rectal temperature was 39.4 °C. Hematological abnormalities included marked thrombocytopenia and mild anemia as well as lymphopenia. No biochemical abnormalities were observed (Table 2). Intraerythrocytic large *Babesia* merozoites were diagnosed again. Therefore, a third subcutaneous injection with imidocarb dipropionate dosed at 9.4 mg/kg BW was given, accompanied by an intramuscular injection of metamizole (40 mg/kg BW) (Table 1).

On day 26, the dog was presented due to tachypnoea, but the general condition and food intake was described as unremarkable by the owner. The general examination was unremarkable besides panting and pale-pinkish mucous membranes. Auscultation of the heart and the lungs was unremarkable. An X-ray was offered to the owners but denied. A mild regenerative anemia and moderate thrombocytopenia were observed in the complete blood count analysis (Table 2). No further therapy regarding the panting was applied.

On day 38, the dog was presented in good condition with unremarkable general examination. No further tachypnoea was detected by the owners but was still positive for *B. canis* in PCR and sequencing, with a higher level of parasitemia of 129,000 *B. canis* parasites/ml blood compared to day 9. Hematological examination revealed mild thrombocytopenia and mild non-regenerative anemia (Table 2). A fourth subcutaneous injection of imidocarb dipropionate at 9.4 mg/kg BW was applied, and doxycycline (10 mg/kg BW once daily p.o.) was added for 10 days (Table 1).

On day 54, 16 days after the fourth imidocarb dipropionate injection, the dog was presented in good condition, and unremarkable behavior was reported by the owners. The general examination, including a rectal temperature of 38.2 °C, was unremarkable. PCR testing was negative and no merozoites were detected in capillary blood smear analysis. Hematological and biochemical examinations including CRP were in the reference range (Table 2). A high *Babesia* spp. antibody level of 65.4 TE was observed (Table 2). Doxycycline was discontinued, and no further medication was applied (Table 1). Until the submission of this report, the dog stayed clinically healthy.

## Discussion

The dog with an acute *B. canis* infection was presented three times due to clinical signs of babesiosis after two imidocarb dipropionate injections at doses of 2.0 and 5.0 mg/kg, with additional prednisolone treatment alongside the second injection. The application of prednisolone most likely contributed to the increasing the level of parasitemia on day 38. At the relapse 12 days after the second imidocarb dipropionate injection, its dose was increased to 9.4 mg/kg BW. Furthermore, the dog showed a persistent moderate to marked thrombocytopenia and mild anemia. After discontinuing prednisolone and another injection of imidocarb dipropionate at 9.4 mg/kg BW on day 38, the dog normalized in behavior, clinical signs, and complete blood count analysis, and *Babesia* PCR testing turned negative.

In Germany, imidocarb dipropionate is used off label. The package leaflets of the most commonly used products indicate a single therapeutic dosage of 0.47–0.58 ml/10 kg BW (i.e., 4–5 mg/kg BW) or of 0.25 ml/10 kg (i.e., 0.2.125 mg/kg BW) and a prophylactic dosage of 0.5 ml/10 kg BW (i.e.,4.25 mg/kg). In the USA, the FDA approved a subcutaneous or intramuscular administration of imidocarb dipropionate twice at a dose of 6.6 mg/kg BW, 14 days apart (FDA). The recommended dosage in literature ranges from 5.0 to 7.5 mg/kg BW (Baneth 2018, Birkenheuer 2023). To address these discrepancies, it should be considered that in Europe the drug was launched in France, a country where predominantly less pathogenic *B. canis* strains were found (Carcy et al. 2015), and/or where dogs were predominately pre-immunized due to previous exposure to less pathogenic *B. canis* strains. Therefore, the aim was to reduce the level of parasitemia and not to clear the *B. canis* infection and thus support pre-immunity. This is supported by recent findings that high *Babesia* spp. antibody levels correspond with less severe clinicopathological abnormalities and acute-phase reactions in dogs with acute *B. canis* infections (Hohnhorst et al. 2025). The current situation of different *B. canis* genotypes in Germany remains challenging, as a broad genetic heterogenicity was detected (Helm et al. 2022), and nothing is known about the pathogenicity of these individual strains.

Reports describing treatment failure in acute *B. canis* infections are rare. In another dog in Germany, PCR remained positive even after five injections of imidocarb dipropionate. However, the first two injections were at a low dose of 2.1 mg/kg BW (Weingart et al. 2024). Additionally, prednisolone was applied twice (1.1 mg/kg BW initially followed by 0.7 mg/kg BW per os for 11 days in tapering dose, another 1.4 mg/kg BW four days later). The following three injections of imidocarb dipropionate at 6.0, 7.7 and 7.5 mg/kg BW could also not provide a negative *B. canis* PCR result. After administration of atovaquone/azithromycin, the PCR was negative within five days. This leads to the discussion of whether the dog might have been (co-) infected with small *Babesia* spp., as the atovaquone/azithromycin treatment is recommended for small *Babesia* spp. infections. In one study in immunocompromised dogs infected with a large unnamed *Babesia* sp., one dog responded to atovaquone/azithromycin (Sikorski et al. 2010). Drug resistance against imidocarb dipropionate is not yet proven in dogs with acute *B. canis* infections and e.g. related point mutations and/or variations in gene copy numbers have not yet been described.

In the present case report, treatment failure can be linked to the initial low dosages of imidocarb dipropionate, as clinical signs disappeared with the two treatments at a dosage of 9.4 mg/kg BW imidocarb dipropionate subcutaneously on days 21 and 38. At the last presentation at the small animal veterinary practice on day 54, no further clinicopathological abnormalities were noted and the PCR provided a negative result. The dog showed a highly positive *Babesia* spp. IgG-antibody level, as expected after exposure to *B. canis*. Evaluation of further serological seropositivity ahead of day 54 was not possible.

Additionally, the application of corticosteroids may also play a significant role in treatment failure. In the presented case, the level of *B. canis* parasitemia dropped from 1,937,000/ml to 5,200/ml blood after the first injection with 2.0 mg/kg BW imidocarb dipropionate. After the application of prednisolone, the level of parasitemia increased to 129.000 *B. canis* parasites/ml blood and the anemia worsened. Most likely, the increase in the level of parasitemia is linked to the application of glucocorticoids due to immunosuppression. In humans diagnosed with malaria, glucocorticoids have been shown to be ineffective for the treatment of severe clinical cases (Hoffman et al. 1988, Warrell et al. 1982). In general, treatment with immunosuppressive dosages of glucocorticoids in canine babesiosis is referred to severe secondary immune-mediated hemolytic anemia and/or to avoid side effects of blood transfusions. Additionally, pulmonary edema as described in dogs infected with *B. canis* (Zygner et al. 2021) may also indicate the use of glucocorticoids. Neither indication was present in the presented case report but a pulmonary edema with tachypnoea on day 26 could not be ruled out for sure as an X-ray was denied by the owners due to the in general good condition and unremarkable auscultation. The uncomplicated course of disease without any specific therapy makes pulmonary edema unlikely in the presented dog. In dogs infected with *Babesia gibsoni*, immunosuppressive treatment may reduce the ability of antibabesial treatment to clear the infection (Birkenheuer 2023).

Due to personal experience, underlined by the findings of the present case report, the authors currently recommend the following treatment protocol against acute *B. canis* infections in non-endemic Central Europe: a first injection of 6.6 mg/kg BW imidocarb dipropionate subcutaneously or intramuscularly, if needed accompanied by supportive care as e.g. intravenous infusions and intensive care. Dogs should be checked by PCR 14 days after treatment. In the case of resolved clinicopathological abnormalities and a negative PCR result, no further treatment is necessary. If the PCR is still positive, an additional injection of 6.6 mg/kg BW imidocarb dipropionate is recommended, followed by another PCR check 14 days later. If remaining positive, *Babesia* species differentiation should be reviewed, as should potential underlying causes for treatment failure mentioned above.

It should be generally considered that imidocarb dipropionate injections may cause parasympathomimetic manifestations such as salivation, nasal discharge, epiphora, and vomiting, which can be alleviated by atropine premedication (Sikorski et al. 2010). In general, a dosage up to 9.9 mg/kg BW is regarded as safe. According to the manufacture’s toxicity study, no effects attributed to imidocarb dipropionate on body temperature, body weight, hematology, most clinical chemistries or gross pathology were recognized at dosages of 2.2, 5.5, 7.7, or 9.9 mg/kg BW. At 9.9 mg/kg BW there was a slight increase in serum alanine aminotransferase and arginine aminotransferase (FDA).

Doxycycline is generally not recommended in acute *B. canis* mono-infections. Therefore, its administration in the presented dog can be critically discussed. In one study, doxycycline as a combination therapy has been reported to reduce the severity of clinicopathological manifestations and to reduce the morbidity/mortality in *Babesia gibsoni* infections (Lin and Huang 2010). Doxycycline is regularly used as prophylaxis against other hematozoa, i.e. *Plasmodium* spp. in humans to prevent malaria (Gaillard et al. 2015). Therefore, doxycycline is considered to have some activity against *Babesia* spp., but controlled studies evaluating efficiency are lacking (Birkenheuer 2023). Its impact on the achievement of a negative PCR result in the presented case report is unclear.

In conclusion, in case of treatment failure in *B. canis* infections defined by a positive PCR after treatment with imidocarb dipropionate, it is recommended to verify that the dog is infected with *B. canis* by species differentiation in PCR testing. Furthermore, dogs with treatment failure should be screened for coinfections and underlying comorbidities, which may complicate the course of an acute *B. canis* infection. Generally, dogs should be treated with a high dosage of imidocarb dipropionate of 6.6 mg/kg BW as approved by the FDA, and the treatment success should be monitored by PCR testing 14 days later. Due to the authors’ personal experience, application of corticosteroids even in non-immunosuppressive dosage should be avoided, especially if blood transfusions are not necessary and/or marked hemolytic anemia is absent. Additionally, as *D. reticulatus* ticks are active year-round as transmitting vectors, year-round ectoparasite prophylaxis with licensed acaricides, even in the winter months, is important to prevent canine babesiosis .

bolded values = parameters outside the reference interval.

## Data Availability

All data generated or analyzed during this study are included in this published article.

## Abbreviations

BW: Bodyweight
CRP: C-reactive protein
DIC: Disseminated intravascular coagulopathy
DNA: Deoxyribonucleic acid
EDTA: Ethylenediaminetetraacetic acid
ELISA: Enzyme linked immunosorbent assay
FDA: Food and Drug Administration
IgG: Immunoglobulin G
MODS: Multi organ dysfunction syndrome
PCR: Polymerase chain reaction
SIRS: Systemic inflammatory response syndrome
TE: Technical units
USA: United States of America
